# A bimodal deep learning framework for cerebral perfusion assessment using synchronous ECG and near-field coupled CBF signals

**DOI:** 10.3389/fbioe.2026.1912951

**Published:** 2026-10-13

**Authors:** Sibei Wu, Junjie Wang, Cai Wan, Gen Li, Zhilong Wang, Jiahao Li, Lin Ning, Siqiao Liu, Jiawen Zhong, Jun Luo, Shuping Yang, Bin Jiang, Shengping Liu, Jingbo Chen, Liang Qiao, Yujie Chen

**Affiliations:** 1 School of Pharmacy and Bioengineering, Chongqing University of Technology, Chongqing, China; 2 College of Artificial Intelligence, Chongqing University of Technology, Chongqing, China; 3 Department of Neurosurgery, Southwest Hospital, Army Medical University, Chongqing, China; 4 College of Biomedical Engineering, Army Medical University, Chongqing, China; 5 Department of Neurology, Southwest Hospital, Army Medical University, Chongqing, China

**Keywords:** bimodal deep learning, cardio‐cerebral coupling, cerebral blood flow (CBF), cerebral perfusion, near-field coupling (NFC)

## Abstract

Accurate real-time assessment of cerebral blood flow (CBF) is crucial for optimizing treatment decisions and improving prognostic outcomes in patients with acute ischemic stroke (AIS). In this study, leveraging the advantage of near-field coupling (NFC) technology in capturing blood flow information from intracranial vessels of different sizes, and in combination with synchronous ECG signals reflecting cardiac rhythm fluctuations, we propose a bimodal deep learning framework to characterize cerebral perfusion-related state changes under postural tilt using synchronous ECG and NFC-based CBF (NFC-CBF) signals. The framework consists of a Multi-Scale Feature Extractor (MSFE) and a Depthwise-Separable Cross-Attention Fusion (DSCAF) module, which work collaboratively to achieve hierarchical temporal representation learning and cross-modal fusion of ECG and NFC-CBF signals. Using synchronous ECG and NFC-CBF data collected from young and middle-aged healthy volunteers, we evaluated the proposed framework in binary and four-class classification tasks to assess its feasibility for cerebral perfusion-related state classification. The results show that NFC-CBF signals can capture CBF changes before and after tilt. Synchronous monitoring of ECG and NFC-CBF provided complementary information on cardiac rhythm and cerebral perfusion-related dynamics. The proposed framework outperforms Bimodal Feature Fusion, ResNet-SE-LSTM, and Cross-Transformer Network in terms of accuracy, F1-score, specificity, precision, and recall, achieving accuracies of 95.95% ± 1.54% and 79.29% ± 2.12% in the binary posture classification and four-class age–posture classification tasks, respectively. Ablation experiments indicate that bimodal modeling provided additional discriminative information compared with single-modality inputs, particularly for the four-class task involving both posture and age. The classification performance achieved after incorporating the DSCAF module is superior to that of other conventional feature fusion strategies. These findings support the feasibility of bimodal ECG and NFC-CBF modeling for cerebral perfusion-related state classification under postural perturbation, while further validation in larger, more diverse, and demographically balanced cohorts, including both patient and non-patient populations, is required before its clinical applicability can be established.

## 1 Introduction

Acute ischemic stroke (AIS) is one of the leading causes of death and long-term disability worldwide, posing a substantial threat to human health and frequently resulting in cerebral tissue necrosis and neurological dysfunction (Sharma and Lee, 2025). Among all stroke cases, AIS accounts for approximately 70% (He et al., 2024a). The dynamic behavior of cerebral blood flow (CBF) directly reflects disturbances in cerebral energy metabolism and the extent of neuronal injury, and is therefore regarded as a key indicator for characterizing the progression of AIS (He et al., 2024b). Accurate and real-time assessment of CBF enables timely tracking of the onset and evolution of cerebral ischemia, which is crucial for optimizing clinical therapeutic decision-making and improving prognostic evaluation in patients with AIS.

At present, CBF assessment mainly relies on imaging-based modalities, including computed tomography (CT), magnetic resonance imaging (MRI), and positron emission computed tomography (PET). Although these techniques provide high spatial resolution and quantitative perfusion information, their high cost, limited availability, and relatively long acquisition cycles make them unsuitable for continuous or real-time bedside monitoring in routine clinical practice (Gaggi et al., 2023; Cerina et al., 2024; Martin et al., 2024). Transcranial Doppler ultrasound (TCD) and near-infrared spectroscopy (NIRS) offer certain advantages for dynamic monitoring; however, TCD is largely limited to flow velocity measurements in major cerebral arteries, while NIRS is constrained by optical attenuation, restricting its ability to reflect deep or whole-brain perfusion states (Manno and Sorond, 2022; Silvaggio et al., 2025). Near-field coupling (NFC) technology has recently attracted increasing attention in vital sign sensing due to its noninvasive, noncontact nature and capability for continuous real-time monitoring (Vo and Trinh, 2024). Previous studies have demonstrated the feasibility of NFC-based approaches for detecting intracranial conditions such as cerebral hemorrhage, edema, and ischemic stroke (Oziel et al., 2019; Barvik et al., 2023). Our group has developed a wearable NFC-based CBF (NFC-CBF) monitoring device capable of simultaneously capturing perfusion changes in both large and small intracranial vessels, and its effectiveness has been validated in healthy volunteers as well as in patients with cerebrovascular diseases, including patients with subarachnoid hemorrhage (Shao et al., 2024; Wang et al., 2025; Xu et al., 2025). Our previous tilt-based work further demonstrated the feasibility of NFC for characterizing dynamic cerebral autoregulation and related cerebrovascular responses under controlled postural perturbation (Shao et al., 2024). Under the same tilt paradigm, NFC responses still exhibited two distinct trends across individuals (Wang and Li, 2024), consistent with the substantial inter-individual variability in cerebral perfusion reported across different populations and pathophysiological states (Burma et al., 2020; Mulser and Moreau, 2023; Feron et al., 2024). Such variability may reduce the generalizability of CBF-only assessment across participants.

AIS not only compromises cerebral perfusion but may also involve impaired cerebral autoregulation (Intharakham et al., 2019). In addition, AIS can precipitate cardiac arrhythmias, reduced heart rate variability (HRV), and other forms of cardiac dysfunction (Lago et al., 2025). In early ischemic stroke, altered HRV and baroreflex sensitivity have been associated with impaired dynamic cerebral autoregulation and larger infarct volumes (Castro et al., 2022). In acute large-hemispheric ischemic stroke, cerebral perfusion has also been associated with cardiac index, indicating a contribution of systemic cardiac hemodynamics to cerebral perfusion (Fuhrer et al., 2017). Recent work on brain–heart interactions in stroke further emphasizes that cerebrovascular impairment and cardiovascular dysregulation should be considered as interdependent processes rather than isolated events (Tan et al., 2025). Cardio-cerebral coupling is mediated through interacting hemodynamic, neural, and humoral pathways. Postural or pathological changes in venous return, cardiac output, and arterial pressure activate baroreflex-mediated sympathetic and parasympathetic adjustments in heart rate and rhythm, while cerebral autoregulation modifies cerebrovascular tone through myogenic, neurogenic, metabolic, and endothelial mechanisms. Humoral factors, including catecholamines, the renin–angiotensin system, and vasopressin, further regulate vascular tone and circulating volume over longer time scales, thereby influencing cerebral perfusion (Claassen et al., 2021; Fan et al., 2024; Candia-Rivera et al., 2026). Accordingly, NFC-CBF serves as the primary modality for characterizing cerebral perfusion-related hemodynamic changes, whereas ECG provides complementary information on cardiac rhythm regulation. Incorporating this cardiac regulatory context can help resolve the ambiguity introduced by inter-individual variability in NFC-CBF responses, thereby improving the discrimination of cerebral perfusion-related physiological states compared with NFC-CBF alone (Castle-Kirszbaum et al., 2022; van Bohemen et al., 2023). Bimodal physiological signal analysis typically relies on handcrafted features extracted in the time, frequency, or time–frequency domains. However, such features may be insufficient to capture nonlinear inter-modal relationships and complex temporal dependencies involved in coordinated cardiovascular and cerebrovascular responses (Liu et al., 2024; Farmani et al., 2025). Recent deep learning studies have therefore progressed from simple feature concatenation toward modality-specific representation learning and attention-based fusion, including ECG–PPG learning for signal reconstruction and atrial fibrillation detection (Ezzat et al., 2024; John et al., 2025), as well as EEG–ECG fusion for physiological-state recognition (Wang and Wang, 2025; Kumar et al., 2026). Multi-scale convolution can capture physiological variations across different temporal ranges (Chen et al., 2025; Reddy C et al., 2025), whereas cross-attention-based fusion can model inter-modal dependencies by selectively emphasizing complementary information and suppressing modality-specific redundancy (Dong and Xie, 2026).

Existing multimodal learning frameworks have demonstrated the effectiveness of multi-scale feature extraction and cross-attention mechanisms for physiological signal analysis. However, most existing methods are designed as general-purpose architectures and do not explicitly consider the heterogeneous temporal characteristics of ECG and NFC-CBF signals, nor the role of ECG as complementary cardiac regulatory context for interpreting cerebral perfusion-related changes. To address these limitations, we propose a bimodal deep learning framework consisting of a Multi-Scale Feature Extractor (MSFE) and a Depthwise-Separable Cross-Attention Fusion (DSCAF) module. The MSFE employs modality-specific multi-scale convolutional branches with kernel sizes designed according to the temporal characteristics of ECG and NFC-CBF signals, enabling physiologically meaningful hierarchical feature extraction. Building upon these representations, the proposed DSCAF introduces depthwise separable convolutions into a shared-query cross-attention architecture, enhancing complementary information exchange between modalities. Postural tilt is an established physiological paradigm for investigating coordinated cardiovascular and cerebrovascular regulation under controlled hemodynamic perturbation (Skytioti et al., 2018; Kato et al., 2022; Mendes Zambetta et al., 2025). Given that these interacting regulatory processes are also relevant to cerebral perfusion regulation after AIS, synchronous ECG and NFC-CBF signals were acquired from healthy volunteers under controlled postural tilt to evaluate the feasibility of the proposed framework for cerebral perfusion-related state classification and to compare it with representative multimodal deep learning models.

## 2 Materials and methods

### 2.1 Synchronized ECG and NFC-CBF signal acquisition

In this study, a synchronized monitoring platform was used to collect ECG and NFC-CBF signals from healthy volunteers during a head-down tilt test, under both the supine and tilted conditions. The platform architecture and the experimental setup are illustrated in Figure 1.

**FIGURE 1:**
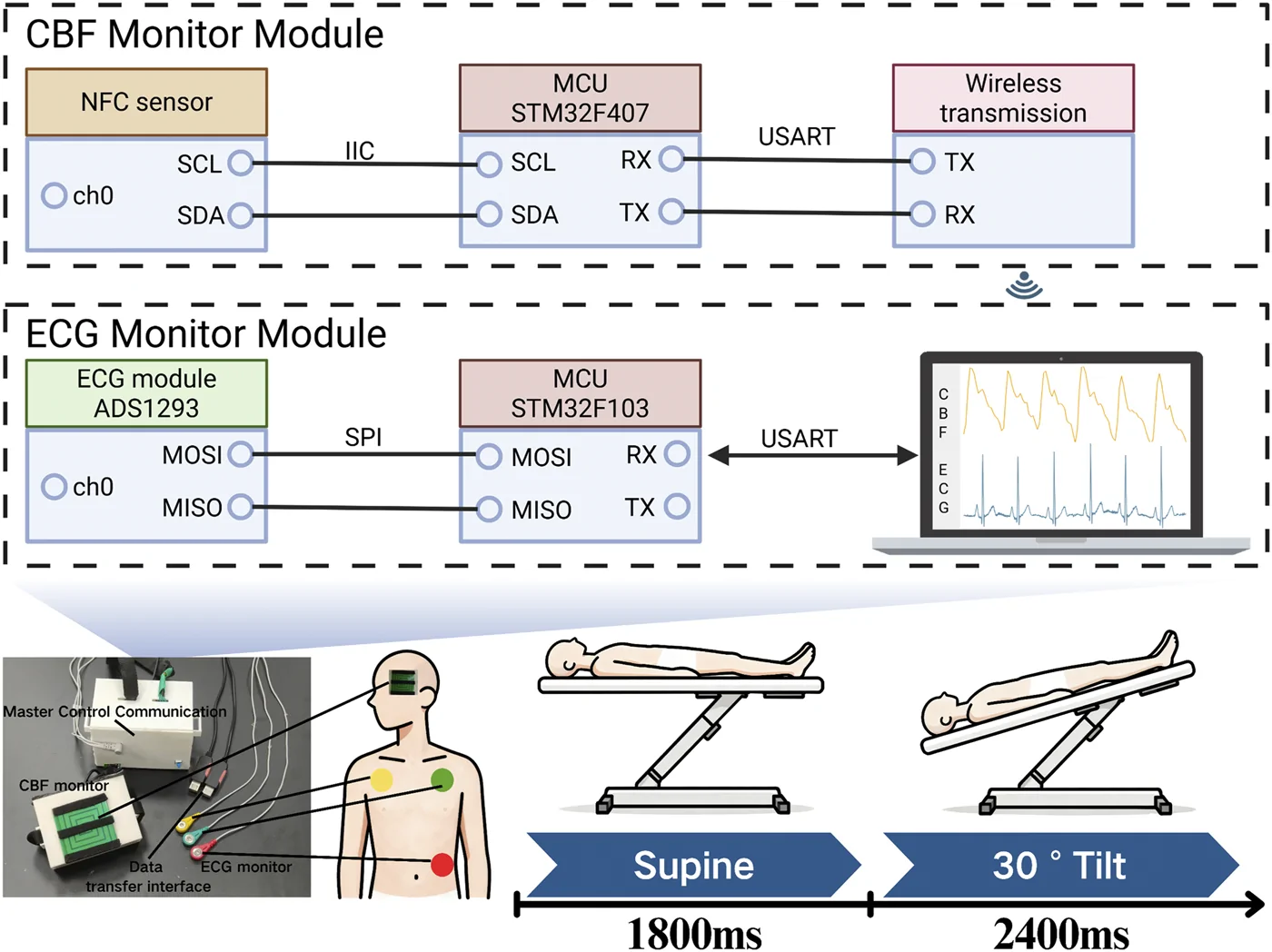
Schematic illustration of the synchronized ECG and NFC-CBF monitoring platform and the experimental signal acquisition setup in healthy volunteers.

The synchronized monitoring platform consisted of two components: an NFC-CBF monitoring module and an ECG monitoring module. The NFC-CBF module comprised an electromagnetic coupling sensor, a signal acquisition unit, a microcontroller, a wireless communication module, and a PC terminal. A dual-layer planar spiral electromagnetic coupling sensor developed in our previous work was employed (Zeng et al., 2023). The signal acquisition unit used a capacitive digital sensor (FDC2214) to convert NFC sensing outputs into a resonant-frequency (RF) signal. A microcontroller (STM32F407) communicated with the acquisition unit via the I^2^C interface, acquired the RF signal at an effective output rate of 18 Hz, and forwarded the data to a wireless module (ESP8266) through a USART interface. The wireless module transmitted the data to a host PC via TCP. For ECG acquisition, an ADS1293-based ECG module communicated with a microcontroller (STM32F103) through SPI and recorded ECG signals at a sampling rate of 144 Hz; the ECG data were then streamed to the PC terminal via a serial connection.

Using the synchronized monitoring platform, we collected data from 20 healthy volunteers, including a young cohort (18–25 years) and a middle-aged cohort (35–55 years), under two postural conditions: supine and 30° tilt. All participants enrolled in this study were male. Before recording, participants rested for 1 min to stabilize their physiological state. Simultaneous ECG and NFC-CBF signals were then recorded for 3 min in the supine position and for 4 min during the 30° tilt condition. All participants had no history of cardiovascular, cerebrovascular, or neurological disorders and refrained from caffeine intake and medication use for 24 h before the experiment. Detailed demographic information is provided in Table 1. The study protocol was approved by the Ethics Committee of Southwest Hospital, Army Medical University (Approval No. 2021KY-5), and written informed consent was obtained from all participants.

**TABLE 1:** Basic information about healthy volunteers.

Number	Group	Height (cm)	Weight (kg)	BMI (kg/m^2^)	Age (years)
01	Young	168	68	24.1	22
02		168	66	23.4	21
03		173	65	21.7	21
04		176	70	22.6	22
05		183	75	22.4	24
06		183	60	17.9	20
07		180	65	20.1	21
08		186	85	24.6	24
09		179	73	22.8	22
10		176	77	24.9	22
11		170	68	23.5	21
12		180	69	21.3	24
13	Middle-aged	178	75	23.7	37
14		170	70	24.2	36
15		175	78	25.5	46
16		173	72	24.1	45
17		170	80	27.7	49
18		172	76	25.7	52
19		165	60	22	42
20		163	62	23.3	37

BMI, was 22.44 ± 1.99 kg/m^2^ in the young group and 24.53 ± 1.74 kg/m^2^ in the middle-aged group (two-sided Welch’s t-test, P = 0.025).

### 2.2 Signal preprocessing and dataset construction

A preprocessing pipeline illustrated in Figure 2 was applied to suppress baseline drift and noise contamination in the acquired ECG and NFC-CBF signals. Baseline drift was mainly induced by physiological and motion-related factors during acquisition, such as respiration, swallowing, and blinking, whereas noise primarily arose from electromyographic activity and power-line interference. For ECG, Butterworth filtering combined with a moving-average kernel was employed for baseline removal and denoising. For NFC-CBF, wavelet threshold denoising was performed using a hybrid strategy that integrates both soft- and hard-threshold filtering. Figure 2A and (B) illustrate the time-domain waveforms and peak–valley detection results before and after preprocessing for ECG and NFC-CBF, respectively. Both modalities exhibited clear periodicity; after smoothing, baseline fluctuations in ECG were effectively reduced while preserving the morphology of the QRS complex and T wave, and the primary peak and dicrotic wave components in NFC-CBF were retained with maximal noise suppression. The resulting preprocessed time series were used for descriptive feature analysis and model training.

**FIGURE 2:**
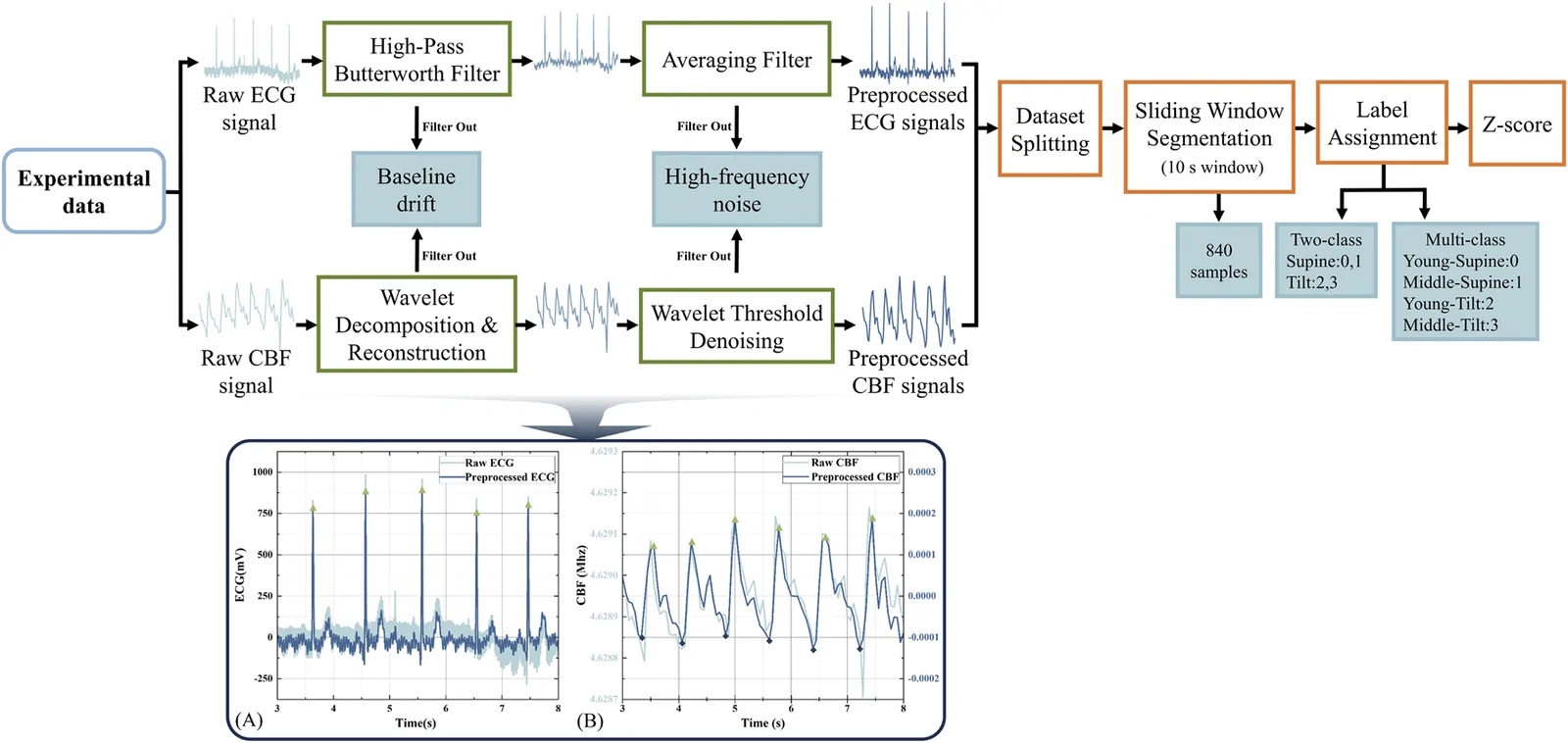
Signal preprocessing and dataset construction pipeline. **(A)** Raw and preprocessed ECG signals. **(B)** Raw and preprocessed NFC-CBF signals.

To characterize individual signal variations under different postural tilt conditions, we extracted an HRV metric from ECG and hemodynamics-derived features from NFC-CBF. For ECG signals, the standard deviation of normal-to-normal intervals (SDNN) was used as the HRV metric to quantify changes in cardiac rhythm before and after the tilt test, as defined in Equation 1 (Wojtanowski et al., 2025).

$$SDNN=\sqrt{\frac{1}{N}\sum_{i=1}^{N}{\left({RR}_{i}-\bar{RR}\right)}^{2}}$$ (1)

Here, *N* represents the number of RR intervals within the window, ${RR}_{i}$ denotes the *i*th heartbeat interval, and $\bar{RR}$ represents the mean RR interval.

R peaks were detected from the preprocessed ECG using the Pan-Tompkins algorithm, and RR intervals were calculated as the time differences between successive R peaks. A sliding-window scheme was then applied to compute SDNN using a 3-min window with a 1-min step, yielding time-varying SDNN estimates over the recording period. For NFC-CBF signals, we extracted the peak-to-valley distance (PVD) and systolic slope (SS) to quantify tilt-induced changes in intracranial blood supply. PVD represents the difference between perfusion influx and outflow within a single cardiac cycle at the measurement site, serving as an indicator of pulsatile blood-volume variation, as defined in Equation 2. SS reflects the rate of blood-volume change per unit time; it incorporates the cardiac ejection duration to account for the influence of cardiac pumping on NFC-CBF dynamics, as defined in Equation 3. To capture the dynamic evolution during the tilt test, both features were computed using non-overlapping 30-s windows. The above features were used for descriptive signal-characteristic

$$PVD=\frac{1}{N}\sum_{i=1}^{N}\left(P_{i}-V_{i}\right)$$ (2)

$$SS=\frac{1}{N}\sum_{i=1}^{N}\frac{P_{i}-V_{i}}{\Delta t_{i}}$$ (3)

Here, *N* represents the number of cardiac cycles within the window. $P_{i}$ and $V_{i}$ denote the peak and its preceding valley of the NFC-CBF signal in the *i*-th cardiac cycle, respectively. $\Delta t_{i}=t_{P,i}‐t_{V,i}$ represents the time interval from $V_{i}$ to $P_{i}$ .

For construction of the deep learning dataset, the preprocessed ECG and NFC-CBF recordings were segmented into non-overlapping 10-s windows, yielding 840 samples. The distribution of samples across the four age-posture classes is provided in Supplementary Table S1. For the experiments, participants were stratified by age group into five mutually exclusive subject-level folds, with all segments from the same participant retained within the same fold. In each iteration, three folds were used for training, one for validation-based learning-rate scheduling and early stopping, and one for testing, with each fold serving as the test set once. Four original age-posture labels were defined: young-supine = 0, middle-aged-supine = 1, young-tilted = 2, and middle-aged-tilted = 3. In the binary task, labels 0/1 were merged as supine and labels 2/3 were merged as tilted; in the four-class task, all four labels were retained. For both experimental protocols, Z-score parameters were calculated separately for ECG and NFC-CBF using the training data only and then applied unchanged to the validation and test data.

### 2.3 Architecture of the bimodal deep learning framework

We propose a bimodal deep learning framework for cerebral perfusion-related state classification under the investigated posture and age conditions, as illustrated in Figure 3. Tailored to the characteristics of ECG and NFC-CBF signals, the framework comprises a Multi-Scale Feature Extractor (MSFE) and a Depthwise-Separable Cross-Attention Fusion (DSCAF) module. The preprocessed ECG and NFC-CBF signals are first fed into the MSFE to learn high-level semantic feature maps at multiple temporal scales. These features are then integrated through the DSCAF to achieve cross-modal fusion. Subsequently, a global average pooling layer is applied to reduce feature dimensionality, followed by a fully connected (FC) layer to further model feature associations. The final predictions are obtained via a Softmax classifier.

**FIGURE 3:**
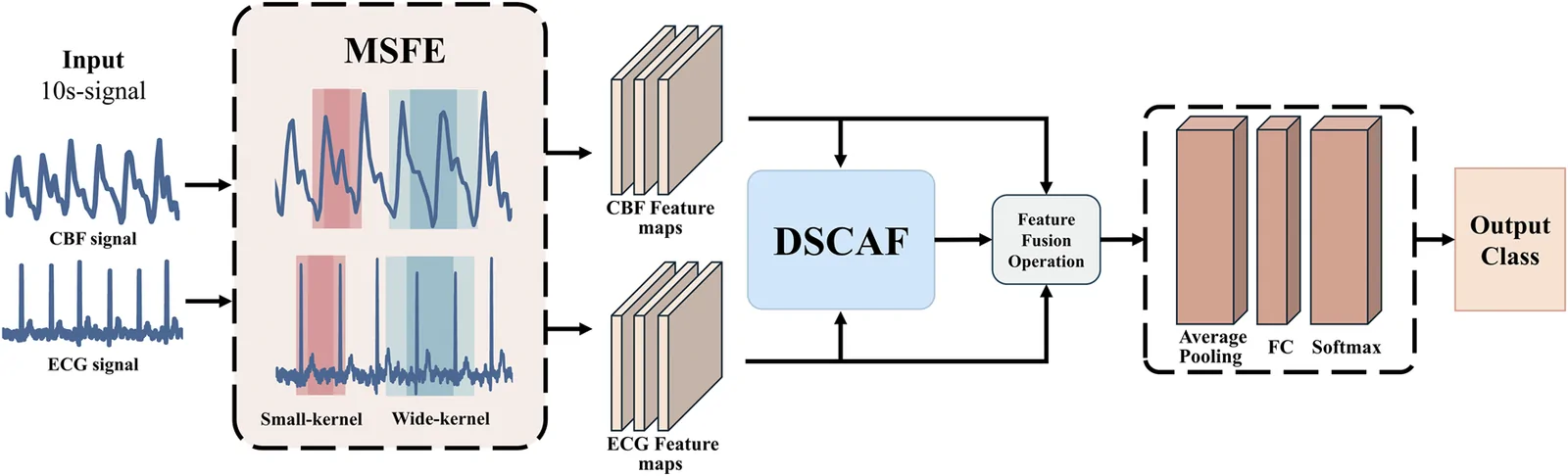
Overall architecture of the bimodal deep learning framework.

#### 2.3.1 MSFE

As shown in Figure 4, the MSFE module adopts a dual-branch multi-scale convolutional architecture, in which parallel 1D convolutional kernels are employed to extract local and cross-cycle features at different temporal scales. Each branch consists of three convolutional layers interleaved with multiple max-pooling operations, and the number of feature channels progressively increases from 128 to 256 and then to 512 as the network depth deepens.

**FIGURE 4:**
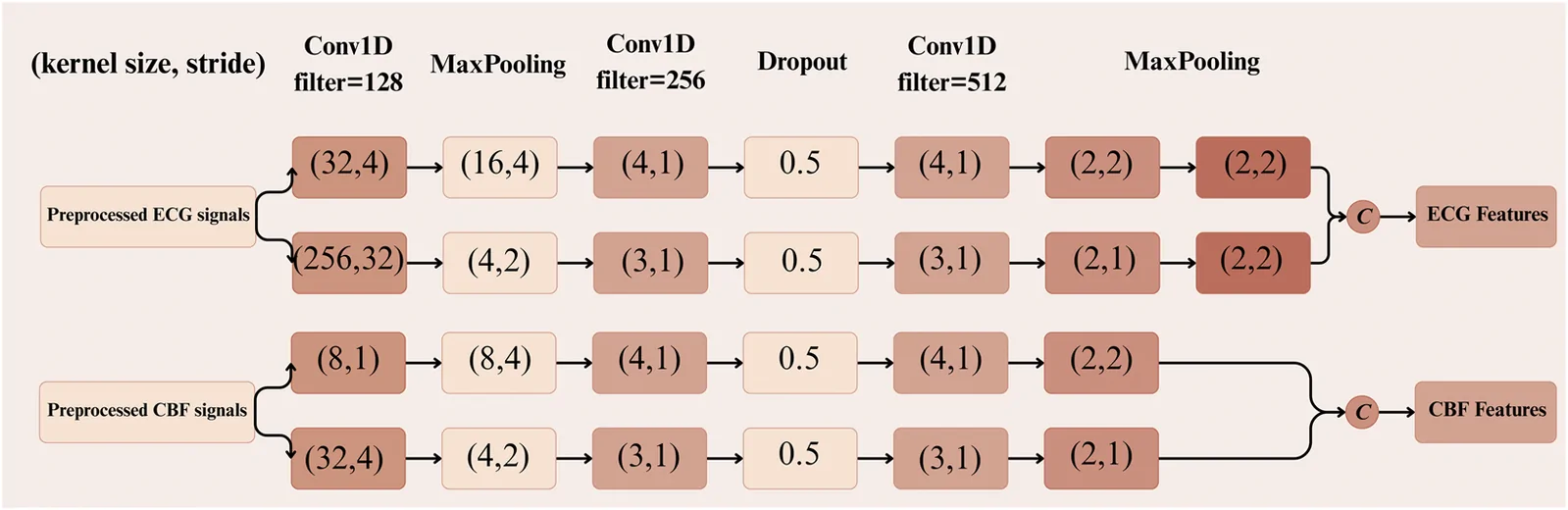
Architecture of the Multi-Scale Feature Extractor (MSFE) module.

Inspired by EEG spectral analysis (Shen et al., 2021), kernel sizes are tailored to physiological temporal intervals: the ECG branch utilizes short (32) and long (256) kernels to capture QRS morphologies and RR-interval rhythms (Reddy C et al., 2025), while the NFC-CBF branch uses kernels of 8 and 32 to model impedance peak-valley details and inter-beat fluctuations (Shao et al., 2024; Wang et al., 2025). After incorporating Dropout to mitigate overfitting, the multi-scale features are concatenated into modality-specific representations (ECG Features and NFC-CBF Features), providing a structurally aligned input for subsequent cross-modal fusion and semantic integration.

#### 2.3.2 DSCAF

As shown in Figure 5, the DSCAF module integrates depthwise separable convolution (DwConv) with a dual-branch cross-attention mechanism to achieve deep semantic interaction and complementary fusion. The MSFE-extracted ECG and NFC-CBF features are first mapped through modality-specific DwConv and fully connected transformations, which are intended to limit parameter growth while preserving distinct modality representations. Unlike a standard Cross-Transformer that relies on full attention operations over high-dimensional token representations, DSCAF constructs two cross-attention pathways using depthwise-separable projections, a shared bimodal query, and modality-specific key-value representations. This design aims to reduce computational redundancy while preserving bidirectional interactions between ECG and NFC-CBF. Replacing standard fully connected layers in the query (Q), key (K), and value (V) generation process, DwConv independently extracts local temporal features before integrating them across channels via pointwise convolution, significantly lowering computational complexity for high-dimensional maps (Bontinck et al., 2024). The module employs a shared query vector derived from concatenated bimodal features, forming two complementary attention pathways: an ECG-guided branch and an NFC-CBF-guided branch. By incorporating element-wise weighted reconstruction and cross-modal residual connections within each branch, the fused outputs are ultimately concatenated and compressed via DwConv to produce a unified bimodal representation that explicitly models cross-modal dependencies.

**FIGURE 5:**
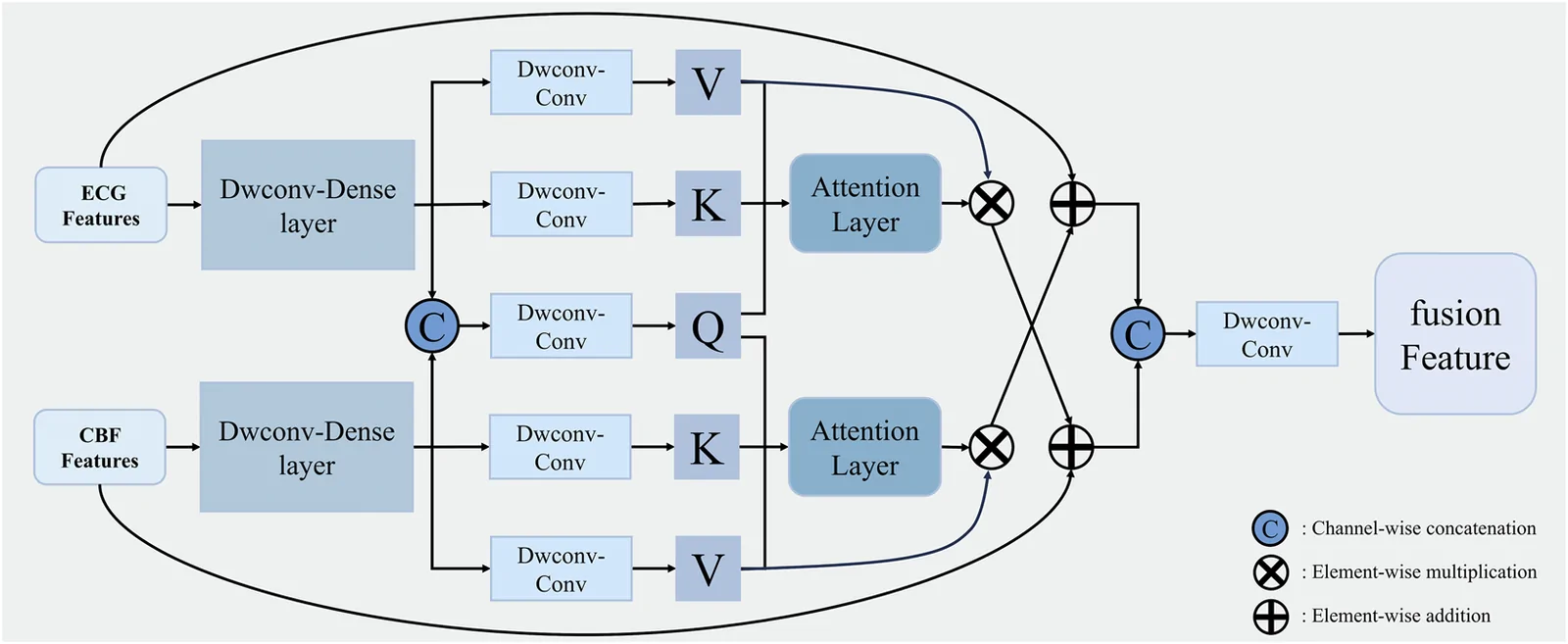
Architecture of the Depthwise-Separable Cross-Attention Fusion (DSCAF) module.

### 2.4 Experimental setup and configuration

To evaluate the proposed bimodal deep learning framework, we designed binary and four-class classification tasks, measuring performance via accuracy, precision, recall, specificity, and F1-score derived from confusion matrices. Model convergence and generalization were monitored through training and validation loss curves. For benchmarking, the framework was compared with three representative models: Bimodal Feature Fusion (Peng et al., 2024), ResNet-SE-LSTM (Li and Gao, 2023), and Cross-Transformer (Pradeepkumar et al., 2024). To ensure a fair comparison, all comparative models used identical 10-s input windows, preprocessing procedures, subject-level fold assignments, hyperparameter-tuning budgets, and evaluation metrics. Ablation studies were performed to compare the individual contributions of ECG and NFC-CBF modalities against the bimodal approach, while also testing the DSCAF module against standard Add, Multiply, and Concat fusion strategies. Accuracy and F1-score were compared using two-sided paired t-tests on matched results from the same five subject-level folds. Within each task and metric, Holm correction was applied separately to the three baseline comparisons and the five ablation comparisons; an adjusted P-value <0.05 was considered statistically significant. Paired mean differences and their 95% confidence intervals were also reported. All models were implemented in PyTorch (v2.1.1) on an NVIDIA RTX 3080 GPU, utilizing the Adam optimizer (initial learning rate of 0.001, β1 = 0.9, β2 = 0.999, and batch size = 512), together with a ReduceLROnPlateau scheduler and early stopping with a patience of 5 epochs.

Integrated Gradients (IG) was applied to representative correctly classified test samples from the binary and four-class tasks to examine the contributions of the two input modalities to the predictions (Sundararajan et al., 2017). Using a zero input—the training-set mean after Z-score standardization—as the baseline, signed attributions were calculated for each ECG and NFC-CBF time point with respect to the true-class output. The absolute attribution values were aggregated within each modality and normalized across modalities to estimate their relative attribution proportions. The top 10% of time points by absolute attribution in each modality were then simultaneously set to zero, and the resulting decrease in true-class probability was calculated.

## 3 Results

### 3.1 Signal characteristics analysis

Figure 6 shows the synchronized ECG and NFC-CBF recordings obtained during the head-down tilt protocol from a representative young participant (Vol. 1) and a representative middle-aged participant (Vol. 13). In Figure 6A, the ECG of Vol. 1 is presented for the supine period, the first 2 min after tilting, and the subsequent 2 min; immediately after tilt onset, the RR interval shortened transiently and then gradually returned toward baseline. Figure 6B indicates that the pulsatile amplitude of the NFC-CBF signal increased promptly after tilting and subsequently decreased to a level that remained higher than that observed in the supine condition. Figures 6C,D present the corresponding results for Vol. 13, who also exhibited posture-related changes in RR intervals and NFC-CBF signals; however, the post-tilt ECG showed irregular rhythm, and both the ECG beats and NFC-CBF pulsatile amplitude varied more mildly, resulting in smaller overall fluctuations.

**FIGURE 6:**
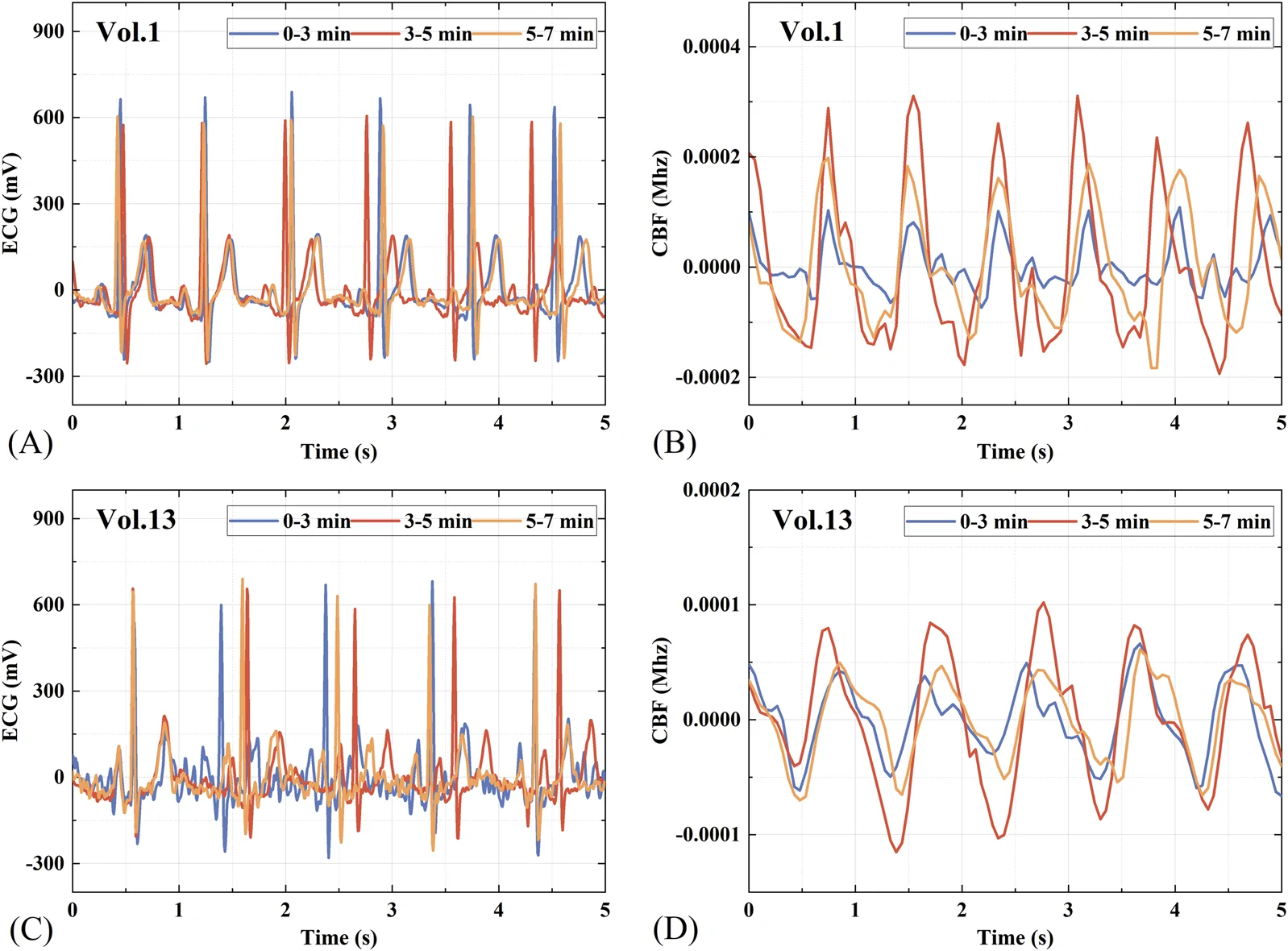
Synchronized ECG and NFC-CBF waveforms during the head-down tilt protocol in representative young and middle-aged participants. **(A,B)** ECG and NFC-CBF signals of Volunteer 1 (young). **(C,D)** ECG and NFC-CBF signals of Volunteer 13 (middle-aged).

The HRV changes observed in the young and middle-aged groups, along with their representative subjects, are shown in Figures 7A,B, respectively. Within the first minute after postural tilt, the mean SDNN increased markedly in both groups, followed by a gradual decline and stabilization over time. The absolute values of SDNN in different age groups differed in terms of the magnitude of change and the recovery process, but they all showed the same phased change characteristics before and after body tilting, indicating that body position changes can stably induce repeatable rhythm regulation patterns in different age groups. Due to gravity, tilting of the body causes changes in the heart’s pulsation pattern and blood distribution, inducing transient changes in HRV and CBF related hemodynamics (Kato et al., 2022; Mendes Zambetta et al., 2025). As the body adapts to the tilted position, these responses gradually stabilize.

**FIGURE 7:**
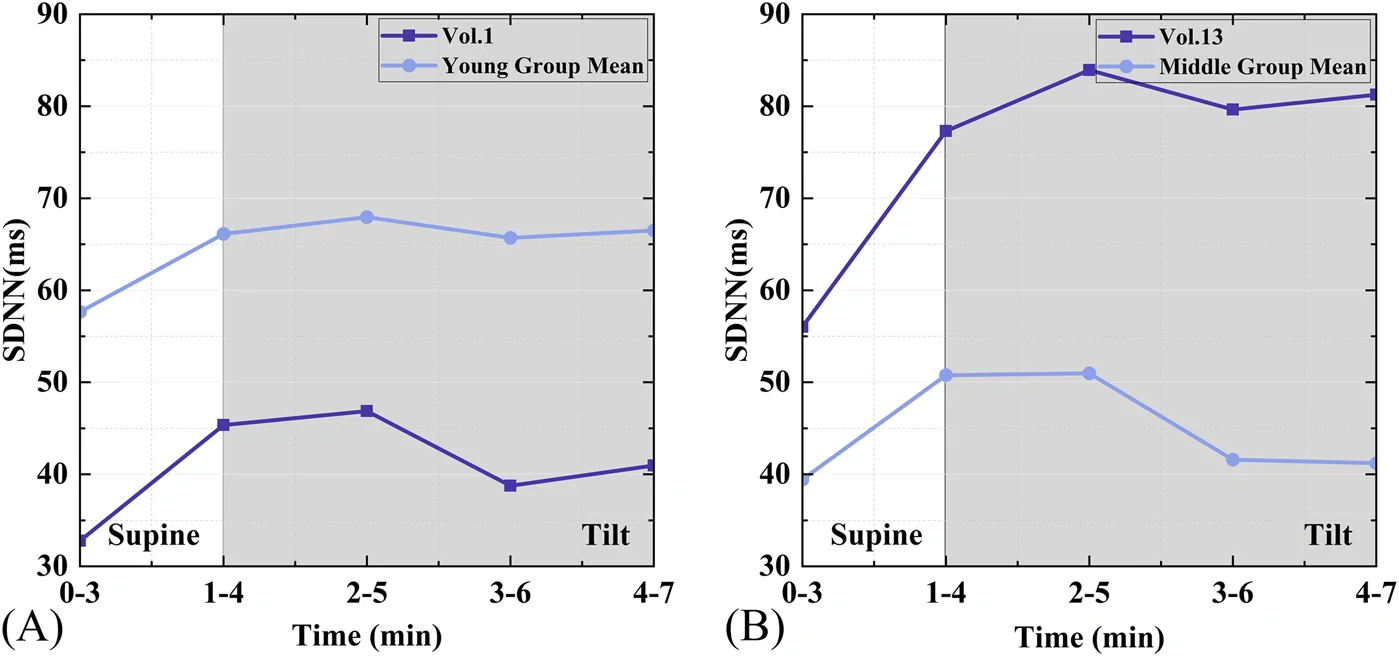
SDNN dynamics during postural tilt in representative participants and the corresponding age groups. **(A)** Volunteer 1 and the young group mean. **(B)** Volunteer 13 and the middle-aged group mean. SDNN, standard deviation of normal-to-normal intervals.

Figure 8 shows the changes in PVD and SS features extracted based on NFC-CBF signals before and after body tilt. Because both increasing and decreasing trends were observed across individuals after tilting, the trajectories are presented separately according to the direction of change, with the mean curves calculated from participants showing the corresponding trend. As shown in Figure 8A, for most young and middle-aged participants, PVD increased immediately after transitioning to the tilted position, followed by a gradual decrease and stabilization, exhibiting a stage-wise variation pattern. Previous validation using the same NFC sensing platform and head-down tilt paradigm showed that NFC-derived VEI and HHC exhibited temporal responses corresponding to TCD-derived CBFV and NIRS-derived rSO_2_, respectively (Shao et al., 2024). By providing multiple parameters associated with intracranial hemodynamic and microcirculatory responses, NFC can provide more comprehensive CBF-related information than either TCD-CBFV or NIRS-rSO_2_ alone. These preceding findings support the physiological basis and reliability of NFC-CBF monitoring for characterizing head-down-tilt-induced cerebral hemodynamic responses.

**FIGURE 8:**
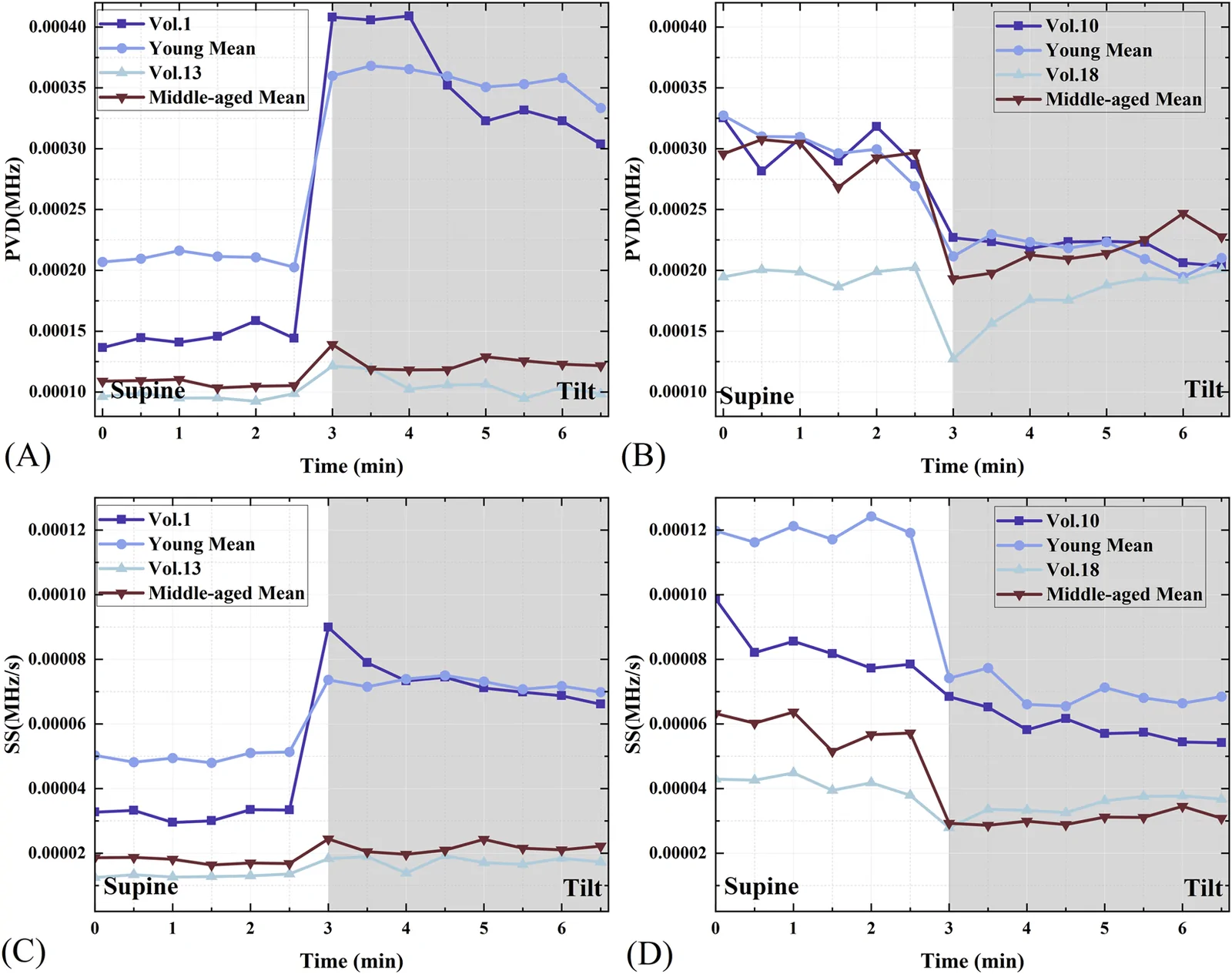
Results of NFC-CBF-derived parameter analysis. **(A)** PVD trends showing increases after tilt in young (n = 9) and middle-aged (n = 5) participants. **(B)** PVD trends showing decreases after tilt in young (n = 3) and middle-aged (n = 3) participants. **(C)** SS trends showing increases after tilt in young (n = 7) and middle-aged (n = 4) participants. **(D)** SS trends showing decreases after tilt in young (n = 5) and middle-aged (n = 4) participants. Mean curves were calculated from participants exhibiting the corresponding trend. PVD, peak-to-valley distance; SS, systolic slope.

However, a subset of individuals in both age groups showed a decrease in PVD after tilting, as illustrated in Figure 8B. In this case, the average PVD trajectories of the two age groups were comparable, whereas the direction and magnitude of changes varied substantially across individuals. Figures 8C,D present the corresponding SS variations before and after tilting, which largely followed trends similar to those observed for PVD. Among participants exhibiting a decreasing SS pattern, the mean SS curves of the young and middle-aged groups differed in both absolute level and dynamic profile. These findings indicate that NFC-CBF–derived features exhibit diverse patterns during postural tilt and are strongly influenced by inter-individual variability. Consequently, relying solely on NFC-CBF signals may be insufficient for robust assessment of cerebral perfusion across individuals.

### 3.2 Analysis of comparative experimental results

To evaluate the overall performance of the proposed framework, it was compared with three representative bimodal time-series models: Bimodal Feature Fusion (Peng et al., 2024), ResNet-SE-LSTM (Li and Gao, 2023), and the Cross-Transformer Network (Pradeepkumar et al., 2024). The results obtained from five-fold cross-validation are summarized in Table 2 and 3.

**TABLE 2:** Comparative results of five-fold cross-validation for the binary classification task.

Model	Accuracy	F1-score	Specificity	Precision	Recall
Bimodal feature fusion	66.19% ± 23.43%	0.58 ± 0.24	0.77 ± 0.27	0.70 ± 0.34	0.51 ± 0.21
ResNet-SE-LSTM	93.81% ± 1.43%	0.93 ± 0.02	0.93 ± 0.02	0.91 ± 0.02	0.95 ± 0.02
Cross-transformer network	81.07% ± 1.81%	0.78 ± 0.02	0.82 ± 0.04	0.77 ± 0.03	0.80 ± 0.03
Our	**95.95% ± 1.54%**	**0.95 ± 0.02**	**0.96 ± 0.02**	**0.95 ± 0.02**	**0.96 ± 0.02**

Values are reported as mean ± SD, across the five subject-level cross-validation folds (n = 5). Supine was treated as the positive class; precision, recall, and F1-score were calculated for the positive class, and specificity for the tilted class. Bold values indicate the best performance for each evaluation metric.

**TABLE 3:** Comparative results of five-fold cross-validation for the four-class classification task.

Model	Accuracy	F1-score	Specificity	Precision	Recall
Bimodal feature fusion	47.62% ± 12.51%	0.41 ± 0.27	0.79 ± 0.07	0.42 ± 0.30	0.41 ± 0.22
ResNet-SE-LSTM	75.83% ± 2.87%	0.76 ± 0.02	0.91 ± 0.02	0.77 ± 0.03	0.75 ± 0.03
Cross-transformer network	70.35% ± 3.13%	0.69 ± 0.03	0.89 ± 0.02	0.71 ± 0.03	0.70 ± 0.04
Our	**79.29% ± 2.12%**	**0.79 ± 0.02**	**0.93 ± 0.01**	**0.80 ± 0.02**	**0.79 ± 0.02**

Values are reported as mean ± SD, across the five subject-level cross-validation folds (n = 5). Precision, recall, specificity, and F1-score are macro-averaged across the four classes. Bold values indicate the best performance for each evaluation metric.

In the binary classification task, the proposed framework achieved the best performance across all evaluation metrics, with an average accuracy of 95.95% ± 1.54%, representing an improvement of approximately 2.1% over the second-best competitor, ResNet-SE-LSTM. The corresponding F1-score and recall were 0.95 ± 0.02 and 0.96 ± 0.02, respectively, indicating balanced discrimination between the supine and tilted samples. In contrast, the Bimodal Feature Fusion model exhibited overall inferior performance with relatively large standard deviations, suggesting limited robustness, while the Cross-Transformer Network achieved intermediate results that were still markedly lower than those of the proposed framework. For the four-class classification task, the proposed framework attained an accuracy of 79.29% ± 2.12%, outperforming the strongest baseline, ResNet-SE-LSTM, by approximately 3.4%. All performance metrics remained at a relatively high level, demonstrating that the proposed framework maintains stable and reliable performance when jointly classifying age groups and postural states. As task complexity increased, the performance of conventional models degraded substantially, whereas the proposed framework preserved strong discriminative capability.

Table 4 presents the fold-wise paired statistical comparisons between the proposed framework and the baseline models. In the binary task, the proposed framework yielded positive improvements over all baseline models in both accuracy (mean paired difference: 0.021-0.298) and F1-score (0.024-0.373). All corresponding 95% CIs excluded zero, with Holm-adjusted P-values of ≤0.038 for accuracy and ≤0.021 for F1-score. In the four-class task, the accuracy improvements ranged from 0.035 to 0.317 and remained significant across all baseline comparisons after Holm correction (adjusted P ≤ 0.009). The F1-score improvements also consistently favored the proposed framework, ranging from 0.035 to 0.381, with all unadjusted 95% confidence intervals excluding zero. Notably, ResNet-SE-LSTM was the closest-performing baseline to the proposed framework in terms of accuracy, yet the corresponding improvements of 0.021 and 0.035 remained statistically significant in the binary and four-class tasks, respectively.

**TABLE 4:** Fold-wise paired statistical comparisons between the proposed framework and baseline models.

Task	Comparison	ΔAccuracy Proposed-baseline (95% CI)	Holm-adjusted P (Accuracy)	ΔF1-score Proposed-baseline (95% CI)	Holm-adjusted P (F1-score)
Binary	Proposed vs. Bimodal feature fusion	0.298 (0.026, 0.570)	0.038	0.373 (0.091, 0.655)	0.021
Binary	Proposed vs. ResNet-SE-LSTM	0.021 (0.012, 0.031)	0.008	0.024 (0.012, 0.036)	0.011
Binary	Proposed vs. Cross-transformer network	0.149 (0.141, 0.156)	<0.001	0.169 (0.154, 0.184)	<0.001
Four-class	Proposed vs. Bimodal feature fusion	0.317 (0.166, 0.467)	0.009	0.381 (0.055, 0.706)	0.063
Four-class	Proposed vs. ResNet-SE-LSTM	0.035 (0.015, 0.054)	0.009	0.035 (0.002, 0.068)	0.063
Four-class	Proposed vs. Cross-transformer network	0.089 (0.052, 0.127)	0.008	0.104 (0.061, 0.147)	0.007

Δ values are paired mean differences across the five matched subject-level folds (n = 5). Statistical comparisons used two-sided paired t-tests, with unadjusted 95% CIs, calculated from the five fold-wise differences using the Student’s t distribution. Holm correction was applied separately within each task and metric across the three baseline comparisons; adjusted P < 0.05 was considered significant.

To further visualize the stability and robustness of the proposed framework across different folds, the performance distributions obtained from five-fold cross-validation are presented as boxplots in Figures 9A,B for the binary and four-class tasks, respectively. In the binary classification task, the proposed framework showed consistently high central performance and relatively compact distributions across the evaluation metrics, and no prominent outliers were observed, indicating strong stability and robustness under different training splits. In contrast, the Bimodal Feature Fusion model showed the largest performance variability, while the ResNet-SE-LSTM and Cross-Transformer Network achieved relatively more concentrated distributions, but with median performance still lower than that of the proposed framework. For the four-class task, the dispersion of ResNet-SE-LSTM, the Cross-Transformer Network, and the proposed framework generally increased, reflecting the greater difficulty of the age-posture combination classification problem. Nevertheless, the proposed framework consistently maintained relatively compact distributions and higher median values on key metrics, demonstrating robust and stable performance in the more complex four-class age-posture classification task.

**FIGURE 9:**
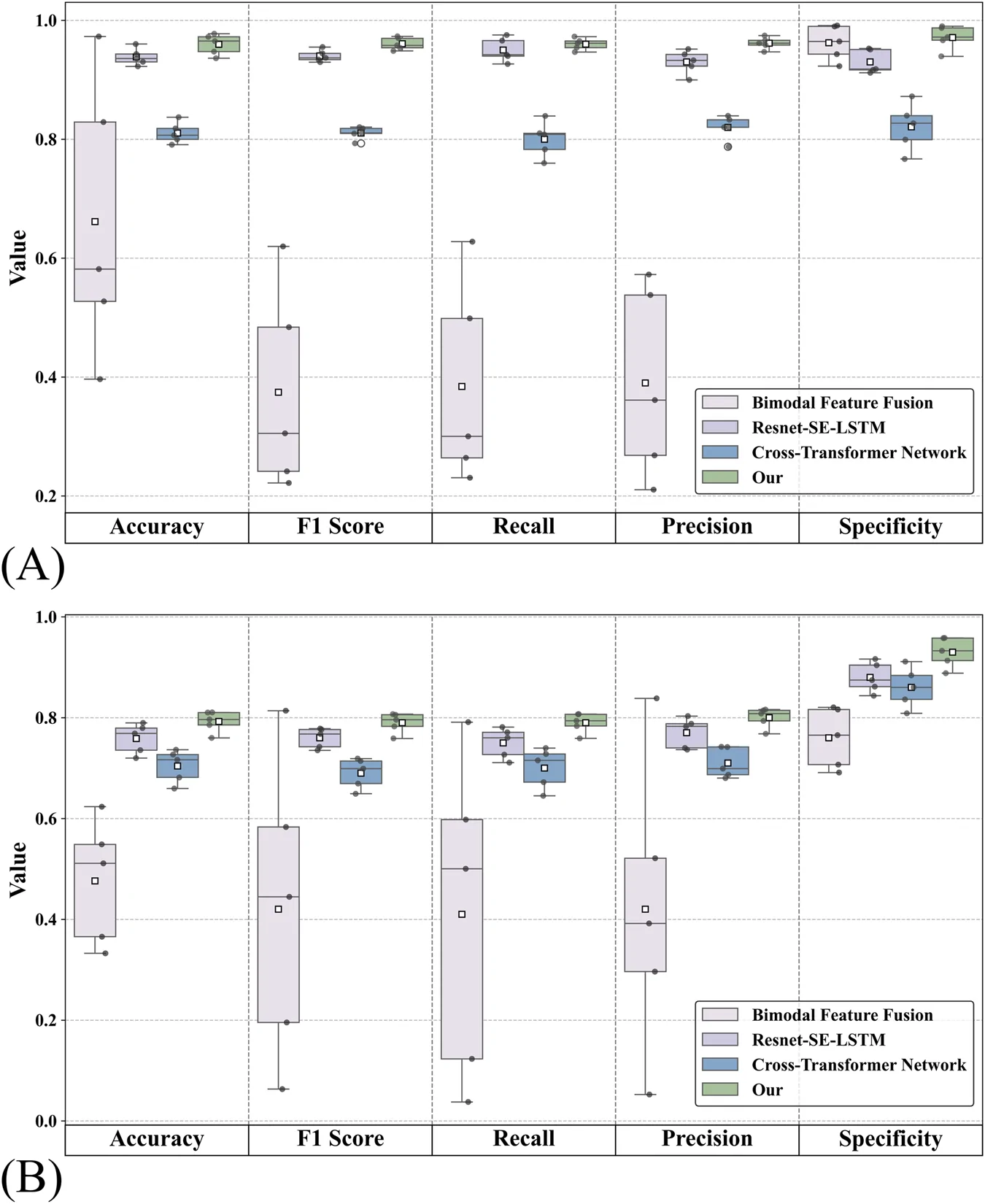
Fold-wise performance stability of different models under five-fold subject-level cross-validation. **(A)** Binary classification task. **(B)** Four-class classification task. Boxplots summarize the five fold-wise values, with the median indicated by the center line, the interquartile range (IQR) by the box, 1.5 × IQR by the whiskers, the mean by the white square, and individual folds by the overlaid points.

### 3.3 Analysis of classification performance

Figure 10 illustrates the classification performance of the proposed framework under subject-level five-fold cross-validation, including pooled out-of-fold confusion matrices, ROC curves, and loss trajectories for the binary and four-class classification tasks. As shown in Figure 10A, the proposed framework achieved high discriminative accuracy for posture classification, with recognition accuracies of 95.56% for the supine condition and 96.25% for the tilted condition, yielding an overall accuracy of 95.95%, consistent with the fold-wise accuracy of 95.95% ± 1.54%. The confusion matrix showed comparable recall rates for the two classes, indicating balanced overall recognition performance across the two postural states. The corresponding ROC curve is shown in Figure 10B, with an AUC of 0.973. The curve lies well above the diagonal reference line, demonstrating strong separability in the binary classification task. As illustrated in Figure 10C, both training and validation losses decreased rapidly with increasing epochs, and the validation curve remained stable without noticeable oscillations, indicating no evident overfitting and suggesting good convergence and stability within the present cohort.

**FIGURE 10:**
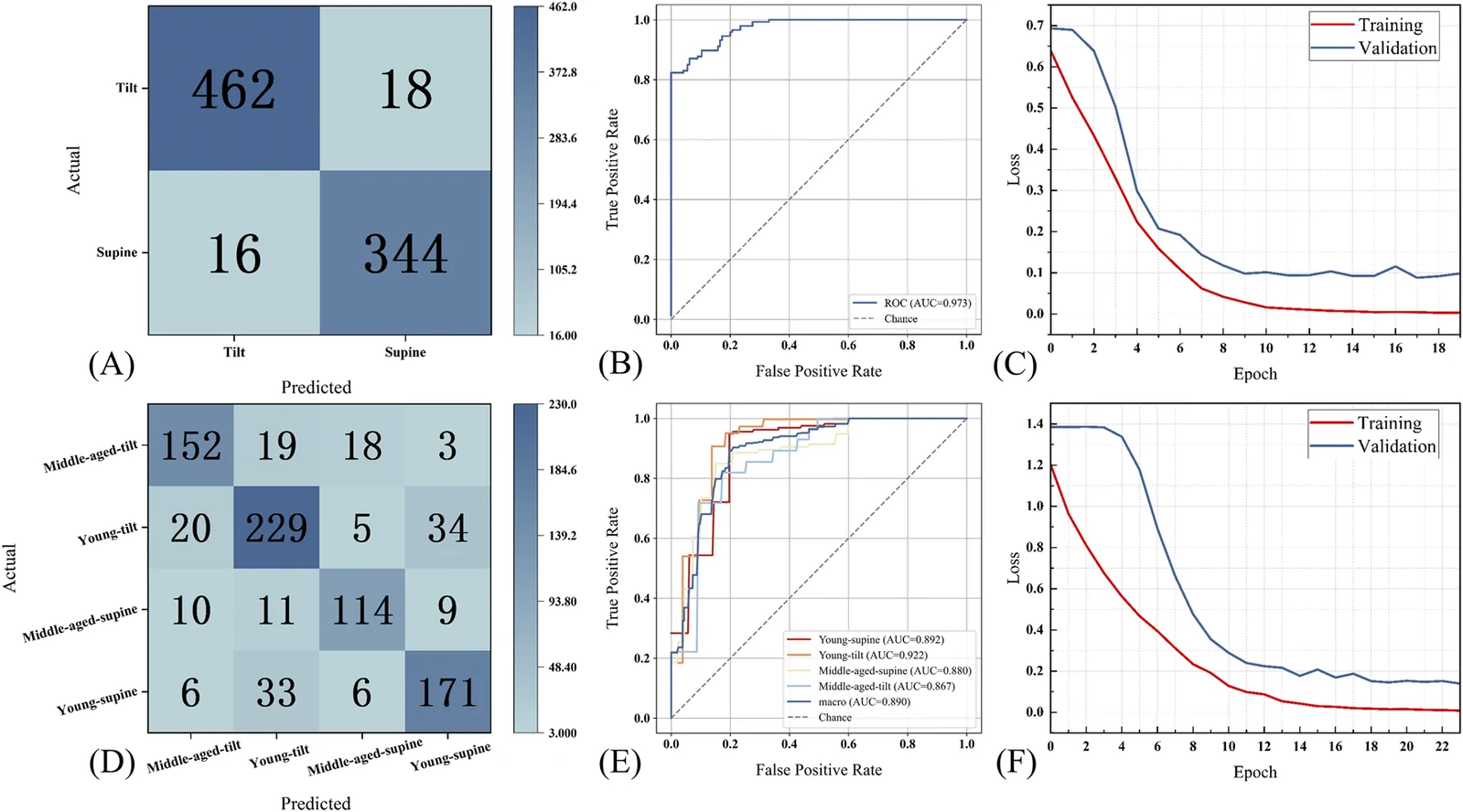
Classification performance of the proposed framework under subject-level five-fold cross-validation. **(A)** Pooled out-of-fold confusion matrix for the binary classification task. **(B)** ROC curve for the binary classification task. **(C)** Training and validation loss curves for the binary classification task. **(D)** Pooled out-of-fold confusion matrix for the four-class classification task. **(E)** ROC curves for the four-class classification task. **(F)** Training and validation loss curves for the four-class classification task. The pooled confusion matrices were obtained by aggregating the test predictions from all five folds. ROC, receiver operating characteristic.

As shown in Figure 10D, the proposed framework maintained relatively high accuracy in the four-class task when distinguishing combined age–posture conditions, achieving an overall accuracy of 79.29%. The class-specific recalls were 79.17% for middle-aged tilted, 79.51% for young tilted, 79.17% for middle-aged supine, and 79.17% for young supine samples. The pooled confusion matrix showed broadly comparable recognition patterns for the young and middle-aged groups. Fold-wise analysis showed that the age-stratified four-class recalls differed by only 0.69 percentage points between the young and middle-aged groups (79.51% ± 2.30% vs. 78.82% ± 2.04%). Class weighting did not improve accuracy, F1-score, or balanced accuracy (Supplementary Table S3). Most errors occurred between different postural states within the same age group, whereas confusion across age groups was comparatively limited. The corresponding ROC curves are presented in Figure 10E, where the class-wise AUC values ranged from 0.867 to 0.922, and the macro-average AUC reached 0.890. Although lower than the binary-task AUC, the macro-average AUC indicated that the framework retained useful class separability when age- and posture-related differences were considered simultaneously. As shown in Figure 10F, the training and validation loss curves exhibited consistent downward trends and gradually stabilized in later epochs, suggesting good convergence behavior and generalization performance for the multi-class task.

### 3.4 Ablation study results

The ablation results are summarized in Table 5 and 6. In the binary classification task, the single-modality baselines showed that NFC-CBF alone achieved substantially higher accuracy (92.74% ± 2.64%) than ECG alone (64.76% ± 5.06%). A similar trend was observed in the four-class task, where the NFC-CBF-only model reached an accuracy of 67.38% ± 6.54%, outperforming the ECG-only model (56.19% ± 6.22%). Overall performance decreased when age was incorporated into the label space, indicating that a single modality is insufficient to simultaneously capture the multidimensional differences associated with both posture and age.

**TABLE 5:** Ablation results of single-modality inputs and different fusion strategies for the binary classification task.

Modality	Fusion methods	Accuracy	F1-score	Specificity	Precision	Recall
ECG	-	64.76% ± 5.06%	0.62 ± 0.06	0.62 ± 0.03	0.57 ± 0.05	0.69 ± 0.09
NFC-CBF	-	92.74% ± 2.64%	0.92 ± 0.03	0.92 ± 0.02	0.90 ± 0.02	0.94 ± 0.04
ECG + NFC-CBF	Add	93.33% ± 2.68%	0.92 ± 0.03	0.93 ± 0.02	0.91 ± 0.03	0.93 ± 0.04
	Multiply	94.05% ± 2.56%	0.93 ± 0.03	0.94 ± 0.02	0.92 ± 0.03	0.94 ± 0.03
	Concat	93.93% ± 1.90%	0.93 ± 0.02	0.94 ± 0.02	0.92 ± 0.02	0.94 ± 0.03
	DSCAF	**95.95% ± 1.54%**	**0.95 ± 0.02**	**0.96 ± 0.02**	**0.95 ± 0.02**	**0.96 ± 0.02**

Values are reported as mean ± SD, across the five subject-level cross-validation folds (n = 5). Supine was treated as the positive class; precision, recall, and F1-score were calculated for the positive class, and specificity for the tilted class. Bold values indicate the best performance for each evaluation metric.

**TABLE 6:** Ablation results of single-modality inputs and different fusion strategies for the four-class classification task.

Modality	Fusion methods	Accuracy	F1-score	Specificity	Precision	Recall
ECG	-	56.19% ± 6.22%	0.56 ± 0.06	0.85 ± 0.02	0.56 ± 0.06	0.56 ± 0.06
NFC-CBF	-	67.38% ± 6.54%	0.70 ± 0.07	0.88 ± 0.02	0.76 ± 0.07	0.66 ± 0.07
ECG + NFC-CBF	Add	74.88% ± 2.87%	0.73 ± 0.03	0.92 ± 0.01	0.73 ± 0.04	0.73 ± 0.03
	Multiply	74.52% ± 2.36%	0.72 ± 0.04	0.91 ± 0.01	0.72 ± 0.04	0.73 ± 0.05
	Concat	73.93% ± 3.13%	0.71 ± 0.03	0.91 ± 0.02	0.72 ± 0.03	0.73 ± 0.02
	DSCAF	**79.29% ± 2.12%**	**0.79 ± 0.02**	**0.93 ± 0.01**	**0.80 ± 0.02**	**0.79 ± 0.02**

Values are reported as mean ± SD, across the five subject-level cross-validation folds (n = 5). Precision, recall, specificity, and F1-score are macro-averaged across the four classes. Bold values indicate the best performance for each evaluation metric.

The bimodal fusion results indicate that all fusion strategies substantially outperformed the single-modality baselines. In the binary task, the Multiply strategy achieved the best performance among the three conventional fusion methods, with an accuracy of 94.05% ± 2.56%. In the four-class task, Add was the best-performing conventional strategy, with an accuracy of 74.88% ± 2.87%. After incorporating the proposed DSCAF fusion module, the framework achieved accuracies of 95.95% ± 1.54% and 79.29% ± 2.12% in the binary and four-class tasks, respectively, representing improvements of approximately 1.9% and 4.4% over the best conventional fusion strategy.

Table 7 presents the fold-wise paired comparisons used to further quantify the contributions of bimodal modeling and DSCAF. In the binary task, DSCAF improved accuracy by 0.032 compared with NFC-CBF alone (95% CI: 0.017–0.047; adjusted P = 0.017), indicating improved classification of the two posture-induced cerebral perfusion-related states, and by 0.019 compared with Multiply, the best-performing conventional fusion strategy (95% CI: 0.003–0.035; adjusted P = 0.039). The corresponding improvements in F1-score were also statistically significant. In the four-class task, DSCAF improved accuracy by 0.119 compared with NFC-CBF alone (95% CI: 0.053–0.185; adjusted P = 0.015), highlighting the particular importance of the complementary information provided by ECG, and by 0.044 compared with Add, the best-performing conventional fusion strategy (95% CI: 0.023–0.065; adjusted P = 0.014). Overall, DSCAF achieved higher performance across the reported metrics in both tasks. The absolute accuracy improvement was particularly pronounced in the more complex four-class task, which required the simultaneous representation of posture- and age-related differences, supporting the advantage of DSCAF in bimodal feature integration and cross-modal semantic interaction modeling. However, as summarized in Supplementary Table S4, these performance gains were accompanied by higher profiled FLOPs, batch-1 inference latency, and peak inference memory than those of conventional fusion strategies, whereas the parameter count and model size remained nearly unchanged and the absolute inference latency remained below 3 m.

**TABLE 7:** Fold-wise paired statistical comparisons between the DSCAF configuration and alternative ablation configurations.

Task	Comparison	ΔAccuracy DSCAF − comparison (95% CI)	Holm-adjusted P (Accuracy)	ΔF1-score DSCAF − comparison (95% CI)	Holm-adjusted P (F1-score)
Binary	DSCAF vs. ECG only	0.312 (0.268, 0.356)	<0.001	0.328 (0.271, 0.386)	<0.001
Binary	DSCAF vs. NFC-CBF only	0.032 (0.017, 0.047)	0.017	0.036 (0.017, 0.055)	0.024
Binary	DSCAF vs. Add	0.026 (0.007, 0.045)	0.039	0.030 (0.007, 0.053)	0.042
Binary	DSCAF vs. Multiply	0.019 (0.003, 0.035)	0.039	0.022 (0.003, 0.040)	0.042
Binary	DSCAF vs. Concat	0.020 (0.009, 0.031)	0.022	0.023 (0.010, 0.037)	0.026
Four-class	DSCAF vs. ECG only	0.231 (0.172, 0.290)	0.002	0.233 (0.177, 0.289)	0.002
Four-class	DSCAF vs. NFC-CBF only	0.119 (0.053, 0.185)	0.015	0.089 (0.024, 0.154)	0.057
Four-class	DSCAF vs. Add	0.044 (0.023, 0.065)	0.014	0.065 (0.003, 0.128)	0.061
Four-class	DSCAF vs. Multiply	0.048 (0.026, 0.069)	0.014	0.076 (0.012, 0.139)	0.061
Four-class	DSCAF vs. Concat	0.054 (0.022, 0.085)	0.015	0.084 (0.039, 0.129)	0.026

Δ values are paired mean differences across the five matched subject-level folds (n = 5). Statistical comparisons used two-sided paired t-tests, with unadjusted 95% CIs, calculated from the five fold-wise differences using the Student’s t distribution. Holm correction was applied separately within each task and metric across the five ablation comparisons; adjusted P < 0.05 was considered significant.

The IG results for representative correctly classified test samples are shown in Supplementary Figure S1. In the binary example, ECG and NFC-CBF accounted for 31.2% and 68.8% of the total absolute attribution, respectively, indicating that this prediction relied primarily on NFC-CBF information while also using the ECG input. In the four-class example, the attribution was more evenly distributed between ECG and NFC-CBF, at 43.1% and 56.9%, respectively. Simultaneously masking the time points with the highest 10% absolute attribution within both modalities reduced the true-class probability by 0.752 and 0.675 in the binary and four-class examples, respectively. These results indicate that specific time points from both modalities contributed to the corresponding bimodal predictions.

## 4 Discussion

Cerebral perfusion is jointly determined by systemic arterial pressure and intracranial pressure and is regulated through the coordinated interplay of multiple mechanisms, including cardiac pulsation, cerebrovascular vasoconstriction–vasodilation, metabolic control, and autonomic baroreflexes (Tzeng and Ainslie, 2014; Claassen et al., 2021). Owing to inter-individual differences in vascular compliance and baroreflex sensitivity, NFC-CBF signals alone are insufficient to comprehensively characterize cerebral perfusion states (Hermann et al., 2024; Tabone et al., 2024). In contrast, ECG signals encode information on cardiac rhythm and autonomic modulation, providing complementary insight into perfusion-related rhythmic dynamics. Joint analysis of ECG and NFC-CBF, therefore, may mitigate variability-related interference and provide a more stable representation of posture-related cerebral perfusion dynamics across participants (Castle-Kirszbaum et al., 2022; Branan et al., 2025). In this study, we integrated synchronously acquired ECG and NFC-CBF time-series signals within a bimodal deep learning framework, embedding cardiac rhythm information and cerebral perfusion–related features into a shared learnable representation space. This design captures complementary temporal information related to cardiac rhythm regulation and cerebral perfusion responses, providing a physiologically grounded functional representation of cardio-cerebral coupling. The MSFE module facilitates the extraction of features associated with both cardiac rhythm modulation and cerebral perfusion characteristics, while the DSCAF module establishes effective cross-modal complementarity, supporting cerebral perfusion-related state classification under postural perturbation.

### 4.1 Physiological interpretation and complementary roles of ECG and NFC-CBF

Pronounced inter-individual variability in resting cerebral blood flow and cerebrovascular responses to postural perturbation has been reported even among healthy individuals (Henriksen et al., 2013; Kato et al., 2022). Previous studies indicate that this variability reflects the combined influence of age, sex, hematocrit, arterial CO_2_, systemic hemodynamics, cerebrovascular resistance and autoregulation, and autonomic and baroreflex regulation, rather than a single anthropometric or physiological factor (Witter et al., 2017; Clement et al., 2018; Claassen et al., 2021). During head-down tilt, cephalad fluid redistribution increases cranial venous and intracranial pressures, while cardiovascular autonomic modulation and cerebrovascular autoregulation jointly shape the resulting cerebral perfusion response (Kato et al., 2022; Mendes Zambetta et al., 2025). Empirically, ECG and NFC-CBF displayed strong temporal synchrony during the transition phase and subsequently entered a new relative steady state. This synchrony is physiologically consistent with rapid autonomic and baroreflex adjustments in cardiac rhythm occurring alongside autoregulatory changes in cerebrovascular tone. Humoral factors may also influence perfusion regulation over longer time scales; however, autonomic activity and circulating mediators were not directly measured in the present study. These interacting regulatory pathways are also relevant to AIS, where altered HRV and baroreflex sensitivity have been associated with impaired dynamic cerebral autoregulation (Castro et al., 2022), while cardiac index has been associated with cerebral perfusion (Fuhrer et al., 2017). By jointly representing cerebral perfusion-related responses and the accompanying cardiac regulatory context, the bimodal framework provides a more physiologically interpretable characterization of cardio-cerebral functional coupling than NFC-CBF alone. HRV metrics derived from ECG showed a consistent pattern across age groups—an initial increase after tilting followed by a gradual decline—indicating that tilt-induced cardiac modulation follows a reproducible regulatory trajectory independent of age. In contrast, the NFC-CBF-derived parameters PVD and SS exhibited substantial inter-individual variability. Accordingly, the heterogeneous PVD and SS responses observed in the present study are physiologically consistent with the multifactorial and individually variable regulation of CBF during postural perturbation. The physiological relevance of these NFC-CBF-derived responses is further supported by previous studies reporting temporal correspondence between NFC-CBF-derived features and TCD- and NIRS-derived cerebrovascular measurements, as well as the sensitivity of PVD and SS to inter-individual response differences during supine-to-tilt testing (Shao et al., 2024; Wang and Li, 2024).

On the one hand, age may represent an important source of the observed variability. Previous studies have reported systematic changes in cerebral blood flow and cerebrovascular resistance across adulthood (Hu et al., 2021; Tomoto et al., 2023). The non-overlapping age ranges, separated by a minimum 10-year interval, provided a physiologically meaningful contrast for examining age-group-related cerebral perfusion patterns. Moreover, the inclusion of healthy middle-aged rather than older adults helped reduce potential confounding from age-related comorbidities, medication use, and overt vascular pathology. Accordingly, the present results support the identification of age-group-related patterns within this young-middle-aged comparison, rather than characterization of cerebral perfusion changes across the entire adult lifespan. Age markedly influences vascular compliance, baroreflex sensitivity, and autonomic regulatory capacity (Feron et al., 2024). Following tilt, gravitational blood redistribution alters arterial and intracranial pressures and consequently changes cerebral perfusion pressure and vascular loading. Consequently, the magnitude of NFC-CBF changes differed considerably between age groups; younger participants, with greater vascular compliance, showed larger NFC-CBF amplitude variations and stronger phase synchrony between NFC-CBF waveforms and the ECG QRS complex than middle-aged participants. On the other hand, because intracranial blood supply is modulated by multiple interacting factors, individuals within the same age group exhibited two distinct trends in NFC-CBF-derived parameter changes before and after tilting. This observation suggests that NFC-CBF signals and their derived features alone are insufficient as reliable markers for cerebral perfusion-related state discrimination across participants.

Head-down tilt induces cardiac-rhythm adjustments associated with autonomic and baroreflex responses alongside changes in cerebral hemodynamics, and ECG captures these beat-to-beat rhythm dynamics with high temporal resolution (Mendes Zambetta et al., 2025). Within the present feasibility framework, NFC-CBF primarily characterizes cerebral perfusion-related responses, whereas ECG provides complementary information on the accompanying cardiac response to postural regulation. Under the same subject-level five-fold splits, the ECG + NFC-CBF model improved accuracy over NFC-CBF alone by 3.2 percentage points in the binary task and 11.9 percentage points in the four-class task (Table 7). These modality-ablation results support the complementary value of ECG-derived cardiac-rhythm information for improving the classification of posture-induced cerebral perfusion-related states, with a larger gain in the four-class task jointly defined by age group and postural state. The IG analysis further complemented the modality-ablation findings at the individual-prediction level. In the representative binary example, NFC-CBF accounted for the larger share of the attribution, consistent with its role as the primary source of cerebral perfusion-related information, whereas ECG also contained prediction-relevant temporal regions, consistent with its complementary cardiac-rhythm role. The marked reductions in true-class probability after masking the highly attributed time points further support the relevance of temporally localized information from both modalities to the corresponding predictions.

### 4.2 Classification performance and architectural contributions

The bimodal deep learning framework is built around an end-to-end joint representation paradigm, in which ECG and NFC-CBF time-series signals are synergistically modeled to establish a new representation of brain-heart coupling characteristics. Unlike conventional pipelines that rely on handcrafted features or shallow classifiers, our framework learns multi-scale temporal features directly from the preprocessed ECG and NFC-CBF time-series segments. Because the two modalities encode complementary physiological dimensions, cross-modal interactive fusion provides more stable discriminative information and reduces misclassification driven by inter-individual variability when relying on NFC-CBF alone (Tang et al., 2024). In the binary task, our framework achieved an accuracy of 95.95%, outperforming ResNet-SE-LSTM (93.81%). The training loss converged rapidly, and the validation loss remained stable without obvious signs of overfitting; moreover, the ROC curve was consistently well above the reference line with an AUC of 0.973, indicating stable predictions with high true-positive rates even at low false-positive rates. In the four-class task, the framework reached an accuracy of 79.29%, and the compact distribution across five folds further suggests robust and stable representation learning for the combined age–posture factors. Within the controlled head-down tilt protocol, the supine and tilted conditions represent two experimentally induced cerebral perfusion-related states characterized by cerebral blood-flow redistribution and accompanying cardiovascular and autonomic regulation. In this physiological context, the binary classification performance demonstrates the capability of the proposed framework to recognize controlled cerebral perfusion-related state changes.

Comparative experiments further substantiate the effectiveness of this representation strategy. Among the baseline models, Bimodal Feature Fusion exhibited the lowest mean performance and the greatest fold-to-fold variability, achieving a binary-classification accuracy of 66.19% ± 23.43%. The pronounced dispersion across the subject-level folds may indicate that this model was particularly sensitive to the composition of the limited cohort. Bimodal Feature Fusion relies primarily on direct channel concatenation, which may aggregate redundant or weakly discriminative information and may be insufficient to align the heterogeneous temporal characteristics of ECG and NFC-CBF signals (Li J. et al., 2024). These limitations may partly account for its unstable performance under the present small-cohort setting. The Cross-Transformer Network exhibited more concentrated fold-wise distributions than Bimodal Feature Fusion but achieved lower mean performance than the proposed framework. Its standard high-capacity cross-attention architecture may be less effective in capturing fine-grained local patterns from small-sample, heterogeneous one-dimensional physiological signals. Although ResNet-SE-LSTM offers strong temporal modeling capability, its serial fusion pipeline restricts sufficient inter-modal interaction. In contrast, by jointly leveraging the MSFE and DSCAF modules to capture both fine-grained local patterns and cross-modal dependencies, the proposed framework consistently maintains a stable advantage in both binary and four-class tasks, providing a more robust bimodal modeling pathway for cerebral perfusion-related state classification.

Unlike existing multimodal architectures that directly combine conventional convolutional feature extractors with standard cross-attention mechanisms, the proposed framework is designed specifically for heterogeneous physiological signals. Both MSFE and DSCAF are developed to address the temporal characteristics of ECG and NFC-CBF signals as well as the constraints associated with relatively small physiological datasets. The MSFE module establishes multi-scale representations that are physiologically aligned with ECG and NFC-CBF signals through a dual-branch multi-scale convolutional design, providing a solid temporal feature foundation for bimodal fusion. By jointly employing short- and long-scale convolutional kernels, MSFE captures both local spike-like patterns and cross-cycle rhythmic information, explicitly covering and structurally aligning the time–frequency scale discrepancies of these heterogeneous signals during feature extraction (Wu et al., 2025). As a result, ECG and NFC-CBF are transformed into complementary and temporally aligned high-level semantic representations before fusion. This design is consistent with the characteristic temporal scales of ECG QRS duration and rhythmic modulation (Chen et al., 2025; Reddy C et al., 2025), as well as with the intra-cycle and inter-cycle temporal distributions of NFC-CBF waveforms (Shao et al., 2024; Wang et al., 2025). Functionally, MSFE acts as a physiological filter bank, addressing the limitation of single-scale convolutions that tend to overlook critical temporal structures. In the comparative models, Bimodal Feature Fusion extracts modality-specific features using a single CNN pathway followed by channel concatenation, lacking explicit multi-scale modeling of cross-cycle dependencies. The Cross-Transformer Network provides limited extraction of fine-grained local features, while the serial architecture of ResNet-SE-LSTM constrains the coordination between feature extraction and subsequent inter-modal interaction. In contrast, incorporating MSFE enhances sensitivity to local time-frequency features across multiple temporal scales, while the parallel design reduces parameter complexity and preserves higher specificity in small-sample settings. This enables superior classification performance and overall stability, and allows subsequent fusion stages to focus more effectively on state-consistent changes shared across modalities, thereby mitigating interference from inter-individual variability and local noise and providing a robust foundation for stable discrimination.

The DSCAF module is designed to model cross-modal semantic interactions and complementary information exchange between ECG and NFC-CBF, while reducing the redundancy introduced by conventional fusion strategies (Gahlan and Sethia, 2024; Li J. et al., 2024). It constructs two symmetric cross-modal interaction pathways using depthwise separable projections, shared queries, and modality-specific key–value representations, allowing each modality to incorporate complementary contextual information while preserving its discriminative features (Farmani et al., 2025). Ablation experiments showed that, compared with conventional fusion methods, the results support the effectiveness of DSCAF in utilizing cross-modal temporal dependencies, and the introduction of DSCAF significantly improved accuracy in both the binary and four-class tasks. Although the improvement in the binary task was statistically significant, its absolute magnitude was modest, suggesting that relatively simple posture discrimination could be captured primarily through modality-specific information and conventional feature fusion, leaving limited room for more complex cross-modal interactions. In contrast, the larger improvement in the four-class task suggests that, when posture- and age-related differences need to be represented simultaneously, complementary information and nonlinear temporal dependencies between ECG and NFC-CBF may become increasingly important for distinguishing partially overlapping classes. Compared with the best-performing conventional fusion strategy in each task, DSCAF achieved these performance improvements with almost no change in parameter count or model size and with an increase of less than 5% in training time per epoch. Although its runtime computational requirements increased, the batch-1 inference latency remained below 3 m. Therefore, the use of depthwise separable projections appears to limit parameter growth and model storage requirements, although it cannot eliminate the additional runtime computation and memory required for cross-modal attention (Li Y. et al., 2024; Zhang et al., 2024). Consequently, in the present study, the cost–performance trade-off was more favorable for the four-class task. By preserving modality-specific representations, the DSCAF module uses cross-modal attention to better capture the latent coupling between the signals and enhance information interaction between the two modalities.

### 4.3 Limitations and future directions

Despite the encouraging performance observed in healthy volunteers, several limitations remain. The sample size was relatively small (n = 20), and all participants were male. Consequently, potential sex-related differences in synchronized ECG and NFC-CBF signal characteristics and their effects on the performance of the proposed framework could not be evaluated. BMI was also significantly higher in the middle-aged group than in the young group. Given this baseline difference and the limited sample size, a potential contribution of BMI to the age-group comparison cannot be excluded, and its specific contribution to the observed between-group differences could not be independently evaluated in the present cohort. Moreover, the cohort did not include pathological populations or a broader range of ages. Future studies should therefore validate the framework in larger and more demographically balanced cohorts, with improved balance of BMI and other baseline characteristics, including patients at different stages after acute ischemic stroke, to further assess its clinical applicability and generalizability. A further limitation is that concurrent TCD, NIRS, or arterial blood-pressure measurements were not acquired in the present cohort; therefore, participant-level correspondence between NFC-CBF responses and independent cerebrovascular or hemodynamic references could not be evaluated. Future studies incorporating synchronous reference measurements are required to extend the framework toward quantitative or clinically defined cerebral perfusion assessment.

Additional challenges concern clinical validation and practical deployment. The present study provides proof-of-concept validation of the proposed bimodal framework for classifying posture-induced cerebral perfusion-related states in healthy individuals under controlled physiological perturbation. AIS represents a more complex and dynamically evolving pathological setting, in which cerebral autoregulation, autonomic regulation, vascular compliance, and cardiac function may undergo time-dependent alterations during post-stroke progression, potentially reshaping the temporal coupling between cardiac rhythm and cerebral perfusion. Accordingly, dynamic cardio-cerebral coupling during post-stroke pathophysiological progression requires further investigation and validation in patients with AIS to determine how the cardio-cerebral coupling information captured by the framework relates to AIS-associated cerebral perfusion abnormalities. The current framework primarily focuses on state discrimination, whereas quantitative modeling of these evolving coupling mechanisms has not yet been fully explored. Future work will therefore aim to incorporate longitudinal monitoring in stroke populations to identify stage-specific coupling signatures and to establish quantitative biomarkers that reflect dynamic changes in cardio-cerebral coupling. Although the batch-1 inference latency was below 3 m on the RTX 3080 GPU, this result cannot be directly extrapolated to wearable or edge hardware, and hardware-specific evaluation and lightweight optimization will be required before practical deployment.

Overall, by integrating NFC-CBF signals with ECG-derived cardiac-rhythm information, the proposed bimodal framework demonstrates the feasibility of classifying posture-induced cerebral perfusion-related states. These findings provide a methodological foundation for future studies incorporating independent cerebrovascular references, larger and more diverse cohorts, and longitudinal clinical data to investigate quantitatively or clinically defined cerebral perfusion outcomes.

## Data Availability

The raw data are not publicly available due to participant privacy and ethical restrictions. Aggregated summary data are provided in the Supplementary Material. De-identified data are available from the corresponding author upon reasonable request, subject to institutional approval.
