# Supplementary material for: A bimodal deep learning framework for cerebral perfusion assessment using synchronous ECG and near-field coupled CBF signals
Source: Front Bioeng Biotechnol. 2026 Oct 13;14:1912951. doi: 10.3389/fbioe.2026.1912951 (PMC13710224; doi:10.3389/fbioe.2026.1912951)
Supplement: Supplementary file 1 [file Table1.docx]

Supplementary Table S1. Distribution of samples across the four age–posture classes.

| Class | Participants, n | Windows per participant, n | Total windows, n | Percentage of total windows (%) |
| --- | --- | --- | --- | --- |
| Young–supine | 12 | 18 | 216 | 25.71 |
| Young–tilt | 12 | 24 | 288 | 34.29 |
| Middle-aged–supine | 8 | 18 | 144 | 17.14 |
| Middle-aged–tilt | 8 | 24 | 192 | 22.86 |
| Total | 20 | - | 840 | 100 |

Note: Each participant contributed data under both postural conditions. Therefore, the participant counts across the four classes are not mutually exclusive, and the total of 20 represents the number of unique participants.

Supplementary Table S2. Summary statistics of physiological features across the four age–posture classes.

| Variable | Young–supine | Middle-aged–supine | Young–tilt | Middle-aged–tilt |
| --- | --- | --- | --- | --- |
| Participants, n | 12 | 8 | 12 | 8 |
| SDNN (ms) | 57.66 ± 17.45 | 39.44 ± 15.84 | 66.10 ± 20.93 | 41.40 ± 20.45 |
| PVD $\text{(}\text{×1}\text{0}^{\text{-4}}\text{MHz}\text{)}$ | 2.33 ± 1.20 | 1.77 ± 1.63 | 3.22 ± 1.76 | 1.59 ± 0.81 |
| SS $\text{(}\text{1}\text{0}^{\text{-5}}\text{MHz}\text{·}\text{s}^{\text{-1}}\text{)}$ | 7.89 ± 6.62 | 3.82 ± 4.52 | 7.11 ± 3.82 | 2.62 ± 1.11 |

Note: Values are presented as mean ± standard deviation across participants. SDNN, standard deviation of normal-to-normal intervals; PVD, peak-to-valley distance; SS, systolic slope.

Supplementary Table S3. Four-class classification performance under unweighted and class-weighted training.

| Training strategy | Accuracy | F1-score | Balanced accuracy | YS Recall | MS Recall | YT Recall | MT Recall | Young-group recall | Middle-aged-group recall | Y−M gap |
| --- | --- | --- | --- | --- | --- | --- | --- | --- | --- | --- |
| Unweighted | 79.29 ± 2.12 | 79.42 ± 1.98 | 79.17 ± 2.09 | 79.44 ± 3.61 | 78.89 ± 1.52 | 79.58 ± 1.35 | 78.75 ± 2.72 | 79.51 ± 2.30 | 78.82 ± 2.04 | 0.69 ± 1.17 |
| Class-weighted | 79.05 ± 2.12 | 78.95 ± 2.01 | 79.00 ± 2.00 | 76.85 ± 4.86 | 80.56 ± 5.56 | 78.61 ± 5.23 | 80.00 ± 4.56 | 77.73 ± 3.57 | 80.28 ± 3.79 | −2.55 ± 6.19 |

Note: Values are presented as mean ± SD (%) across the five subject-level folds. YS, young-supine; MS, middle-aged-supine; YT, young-tilt; and MT, middle-aged-tilt. YS Recall, MS Recall, YT Recall, and MT Recall denote the class-specific recall for the corresponding four classes. Young-group recall and middle-aged-group recall were calculated as the macro-average recalls of the YS and YT classes and of the MS and MT classes, respectively. The Y–M gap represents young-group recall minus middle-aged-group recall and is reported in percentage points. The unweighted and class-weighted models were evaluated using identical subject-level folds and training settings, with class weights calculated from the training data of each fold.

Supplementary Table S4. Computational complexity and runtime characteristics of the evaluated fusion configurations.

| Task | Fusion configuration | Parameters  (M) | Profiled FLOPs (MFLOPs/sample) | Model size  (MB) | Training time per epoch (s) | Batch-1 inference latency (ms) | Peak inference memory (MB) |
| --- | --- | --- | --- | --- | --- | --- | --- |
| Binary | Add | 2.5786 | 0.010756 | 9.996 | 4.510±0.019 | 1.493±0.045 | 47.156 |
| Binary | Multiply | 2.5786 | 0.010756 | 9.996 | 4.489±0.016 | 1.460±0.067 | 47.156 |
| Binary | Concat | 2.5788 | 0.001028 | 9.997 | 4.511±0.029 | 1.512±0.036 | 47.163 |
| Binary | DSCAF | 2.5788 | 0.124420 | 9.997 | 4.693±0.022 | 2.833±0.059 | 73.400 |
| Four-class | Add | 2.5788 | 0.011272 | 9.997 | 4.383±0.016 | 1.416±0.033 | 47.163 |
| Four-class | Multiply | 2.5788 | 0.011272 | 9.997 | 4.399±0.024 | 1.421±0.044 | 47.163 |
| Four-class | Concat | 2.5794 | 0.002056 | 9.999 | 4.395±0.012 | 1.461±0.046 | 47.177 |
| Four-class | DSCAF | 2.5794 | 0.125448 | 9.999 | 4.583±0.010 | 2.722±0.070 | 73.414 |

Note: Parameters and model size were calculated for the complete model under each fusion configuration. Profiled FLOPs represent the floating-point operations of profiler-supported operators during one forward pass with a batch size of 1 and do not represent the complete computational workload of the model. Training time and batch-1 inference latency are reported as mean ± standard deviation. Runtime and memory measurements were obtained on an NVIDIA RTX 3080 GPU.
